# Unlocking the anti‐diabetic potential of *Gymnema sylvestre*, *Trigonella foenum‐graecum*, and their combination thereof: An in‐vivo evaluation

**DOI:** 10.1002/fsn3.3685

**Published:** 2023-09-18

**Authors:** Muhammad Kashif, Amar Nasir, Gulzaman, Muhammad Kamran Rafique, Mazhar Abbas, Aziz ur Rehman, Muhammad Riaz, Ghulam Rasool, Andrew G. Mtewa

**Affiliations:** ^1^ Department of Clinical Sciences (Section Clinical Medicine), College of Veterinary and Animal Sciences University of Veterinary and Animal Sciences, Lahore (Jhang Campus) Jhang Pakistan; ^2^ Department of Pathobiology, College of Veterinary and Animal Sciences University of Veterinary and Animal Sciences, Lahore (Jhang Campus) Jhang Pakistan; ^3^ Department of Basic Sciences (Section Biochemistry), College of Veterinary and Animal Sciences University of Veterinary and Animal Sciences, Lahore (Jhang Campus) Jhang Pakistan; ^4^ Department of Allied Health Sciences University of Sargodha Sargodha Pakistan; ^5^ Chemistry Section, Malawi Institute of Technology Malawi University of Science and Technology Limbe Malawi

**Keywords:** anti‐diabetic effect, diabetes, glucose level, *Gymnema sylvestre*, serum biochemistry, *Trigonella foenum‐graecum*

## Abstract

The current study aimed to explore the anti‐diabetic effect of aqueous extracts of *Gymnema sylvestre*, *Trigonella foenum‐graecum* and mixture of both the plants in alloxan‐induced diabetic rabbits. A total of 30 rabbits were grouped into six equal groups as: normal control, diabetic control, diabetic treated with 300 mg/kg body weight (bw) *G. sylvestre*, diabetic treated with 300 mg/kg bw *T. foenum‐graecum*, diabetic treated with 300 mg/kg bw mixture of both the plants and diabetic treated with 500 mg/kg bw metformin for 4 weeks. Diabetes was induced to all the study group animals except normal control by intravenous administration of alloxan monohydrate (80 mg/kg bw). Blood glucose was measured by glucometer and other biochemical parameters were determined through various kit methods. Serum insulin was measured through ELISA kit method. Results showed that both the plants and metformin significantly (*p* < .05) decreased the fasting blood glucose. Hypoglycemic activity of aqueous extract of *G. sylvestre* and metformin was found slightly higher than aqueous extract of *T. foenum‐graecum* and the mixture of both the plants. However, a significant (*p* < .05) rise in insulin secretion was observed in studied plants extract treated rabbits. Serum urea, creatinine, and liver enzymes were found reduced significantly (*p* < .05) in treated rabbits whereas packed cell volume was also returned to normal in treated animals as compared to control group. The study concluded that *G. sylvestre* and *T. foenum‐graecum* extracts have comparable effects with metformin in normalizing the blood glucose level and have more pronounced effect than metformin in restoring the serum biochemical parameters to normal levels. Hence, these plants may be the good alternative medicine in managing the diabetes mellitus.

## Graphical abstract

Methanolic extract of *Gymnema sylvestre* and *Trigonella foenum‐graecum* strongly possessed the anti‐diabetic property. These effects are comparable with the standard drug metformin. The studied plants extract treatment maintained the body weight, decrease the urea and creatinine level, and increases the activity of liver enzymes in diabetic rabbits.

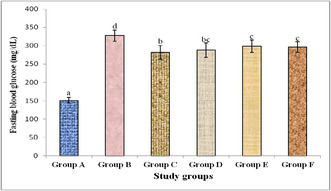

## 1 INTRODUCTION

Diabetes mellitus is a serious metabolic disease characterized by persistent hyperglycemia resulting in impaired insulin resistance, insulin sensitivity, or both due to changes in the metabolism of carbohydrate, protein, and lipid (Laila et al., 2023). According to estimates from the reports of International Diabetes Federation, more than 783 million people will develop diabetes by 2045, with 537 million adults living with diabetes and 124.87 million in India (Kahksha et al., 2023; Sun et al., 2022). Diabetes and its consequences remain a serious medical problem despite the development of hypoglycemic medications (Bint Mustafa et al., 2016; Peter et al., 2018). Synthetic oral hypoglycemic medications can cause significant complications (Kahksha et al., 2023).

There are over 1200 species of plants which have been reported to treat the diabetes mellitus due to their alleged hypoglycemic potential around the world (Salehi et al., 2019). *Gymnema sylvestre* is a plant indigenous to India's central and western regions, as well as tropic zone of Africa and Australia. It is a powerful anti‐diabetic plant that has been used in folk, Ayurveda, and homeopathic medicines for centuries (Khan et al., 2019). Triterpene saponins of the oleanane and dammarene classes are found in *G. sylvestre*. Gymnemic acid and Gymnema saponins are oleanane saponins, whereas gymenasides are dammerene saponins. Following its successful isolation and purification from *G. sylvestre*, all the molecules related to gymnemic acid were recognized as anti‐diabetic (Harshavardhana & Krishna, 2019). The leaves which are rich source of gymnemic acid may reduce hyperglycemia through the mechanisms like; increasing the enzymes activities that are actively involved in glucose utilization, increase in phosphorylation, decreasing the activity of enzymes involved in gluconeogenesis and sorbitol dehydrogenase. It also reduces or delays the intestinal absorption of glucose (Beula et al., 2023). Different civilizations around the world have employed herbal medicines for diabetes prevention and management since long time. Although, gymnema species have been used ethnomedically in treating diabetes in India for many years, scientific evidence for their therapeutic and pharmacological properties have just lately been published. Some gymnema species have been reported to assist in the repair or regeneration of beta cells, which are involved in insulin synthesis and secretion (Khan et al., 2019). *G. sylvestre* leaves aqueous extract decrease the level of blood glucose by rebuilding the islets and beta cells of the pancreas in diabetic rats. In type 2 diabetes mellitus, 400 mg daily dose of *G. sylvestre* aqueous extract lowers the HbA1C level while significantly decreased the insulin demands (Rahman et al., 2022).

*Trigonella foenum‐graecum* (Fenugreek) from Fabaceae family is a valuable medicinal plant. Seeds of this plant are mostly used in Mediterranean, African, and Asian countries as major ingredients in medicine, nutrition, beverages, fragrances, and cosmetics (Fatima et al., 2022). It is used in folk medicine for the treatment of several conditions like diabetes and obesity. It possesses anti‐inflammatory, anti‐oxidant, anti‐microbial, anti‐hyperglycemic, and anti‐hyperlipidemic properties (Nagulapalli Venkata et al., 2017). Pharmacological effects of *T. foenum‐graecum* are attributed due to the presence of a variety of bioactive compounds including flavonoids, polyphenols, saponins, alkaloids, steroids, amino acids, and carbohydrates. Diosgenin saponin is reported as the most bioactive substance of this plant having anti‐oxidative effects and plays significant role for the improvement of diabetic status by various mechanisms (Baset et al., 2020). Diabetic rabbits treated with the oil of fenugreek had considerably low level of lipid peroxidation, creatinine, albumin, and urea (Hamden et al., 2010). WHO recommended to search for anti‐diabetic plants with least cost and lesser side effects. It is due to their ability to protect body cells from diabetic complications and to restore the pancreatic cells that produces insulin (Salehi et al., 2019). Keeping in view the importance of medicinal plants in various ailments (Mtewa, 2017; Akram, 2021; Mtewa et al., 2020; Mtewa & Egbuna, 2021), this study was designed to explore the anti‐diabetic effects of *G. sylvestre* and *T. foenum‐graecum* with metformin in alloxan‐induced diabetic rabbits.

## 2 MATERIALS AND METHODS

### 2.1 Materials

Alloxan Monohydrate were purchased from MP Biomedicals, LLC, Tab Metformin 500 mg purchased from local pharmacy, Glucometer from Capricorn Scientific, spirit and alcohol from local market, Pakistan.

### 2.2 Methods

#### 2.2.1 Collection of plants and preparation of extracts

The leaves of *G. sylvester* and seeds of *T. foenum‐graecum* were purchased from local market of Jhang, Punjab, Pakistan. The leaves of *G. sylvester* were shade dried, ground to powder, and then 100 g powder was soaked in 100 mL distilled water (DW). After 24 h, the extract was filtered and the solvent was evaporated through rotary evaporator to obtain semisolid extract for further analysis (Aralelimath & Bhise, 2012). On the other hand, the seeds of *T. foenum‐graecum* were dried by incubating at 40°C for 48 h and ground to powder form. The 50 g seeds powder was macerated as outlined by Mtewa et al. ( 2018) in 500 mL DW and incubated at 37°C for 36 h. After filtering, filtrate was dried and yellow color residue was obtained (Ghosh et al., 2009).

#### 2.2.2 Phytochemical analysis

The total phenolic contents in aqueous extracts of studied plants were determined by Folin–Ciocalteu reagent method using gallic acid as standard. Briefly, the test extract was mixed with water, warmed and filtered. The 5 mL of filtrate was allowed to react with 1 mL of 5% ferric chloride solution. Dark green or deep blue color showed the presence of tannins and phenols (Waseem et al., 2021). Total flavonoid contents in aqueous extracts of plants were determined by aluminum chloride colorimetric assay using quercetin as the standard (Ayub et al., 2017). Alkaloids were determined by vincristine through spectrophotometer at 565 nm (Akinmoladun et al., 2007). Tannins were estimated by Folin Denis reagent method (Midkiff, 1984). Saponins were determined by gravimetric method (Jorns et al., 1997).

#### 2.2.3 Animals and experimental design

The study plan was dually approved by the Ethics Committee of the University of Veterinary & Animal Sciences, Lahore (No. CVAS/13546 dated 07‐01‐2021) and by the Directorate of Advanced Studies of the same university (DAS/537 dated 15‐06‐2021). A total of 30 rabbits were purchased from the local market weighing 1–1.5 kg and were acclimatized for 1 week in experimental rooms of the College of Veterinary and Animal Sciences, Jhang. Rabbits were divided into six equal groups (*n* = 5) and kept in separate cages at room temperature (25–28°C), and fed green fodder and pellets with ad‐libitum supply of fresh water. Blood glucose and weight were monitored at days 7, 14, 21, and 28. The dose of plants extract as 300 mg/kg body weight (bw) was selected based on literature review. Laila et al. (2023) studied the anti‐diabetic potential of *T. foenum‐graecum* using different dose concentrations and found significant results at 300 mg/kg bw of animals. The grouping of rabbits is given below:

1. *Group A*: Negative control (Receiving DW).
2. *Group B*: Diabetic control (Alloxan‐induced diabetic receiving no treatment).
3. *Group C*: Alloxan‐induced diabetic receiving 500 mg/kg bw Metformin as standard drug for diabetes.
4. *Group D*: Alloxan‐induced diabetic receiving 300 mg/kg bw aqueous extract of *G. sylvestre*.
5. *Group E*: Alloxan‐induced diabetic receiving 300 mg/kg bw aqueous extract of *T. foenum‐graecum*.
6. *Group F*: Alloxan‐induced diabetic receiving 300 mg/kg bw aqueous extract of both plants.

#### 2.2.4 Induction of diabetes

Diabetes was induced in group B, C, D, E, and F by injecting alloxan monohydrate at the dose rate of 80 mg/kg bw after overnight fasting. After 1 week, blood glucose level of all rabbits was measured by using glucometer. Those rabbits with blood glucose level more than 300 mg/dL were considered diabetic and included in the study.

#### 2.2.5 Measurement of weight and fasting blood glucose level

Fasting blood glucose levels was measured using glucometer (Accu Chek Performa®). Glucose levels was observed at day 1 (before treatment), then regularly after every week during the experiment and weight of each rabbit was recorded before diabetes and each week after induction of diabetes using digital electric balance.

#### 2.2.6 Determination of insulin level

Insulin level was measured at 0 day (before treatment) and at end of the experiment. A volume of 3 mL of blood samples were obtained from all the study animals after 4 weeks and insulin level was determined by radio immunoassay using Insulin Enzyme Linked Immunosorbent assay (ELISA) Kit and immunoreactive insulin (free insulin + insulin bound to anti‐insulin antibodies) was measured in serum.

#### 2.2.7 Measurement of liver enzymes, renal function tests and packed cell volume

The activity of liver enzymes including alanine aminotransferase (ALT), aspartate aminotransferase (AST), and alkaline phosphatase (ALP) was measured by kit methods using automated chemistry analyzer according to the manufacturer's instructions (Mahmood et al., 2013). Serum creatinine and blood urea nitrogen levels were also measured by kit methods while packed cell volume (PCV) was determined by automated hematology analyzer.

#### 2.2.8 Statistical analysis

The data were analyzed through one‐way ANOVA using IBM SPSS Statistics 24, followed by post‐hoc analysis. Statistical significance was considered at *p* < .05.

## 3 RESULTS

### 3.1 Phytochemical analysis

Phytochemical analysis of aqueous extracts of both the plants showed various quantities of phytoconstituents, including phenolics, flavonoids, alkaloids, tannins, and saponins and the results are given in Table 1. No marked differences in the contents of most of the phytoconstituents except tannins were observed in aqueous extracts of *G. sylvestre* and *T. foenum‐graecum*. Higher tannin content was found in *G. sylvestre* extract as compared to *T. foenum‐graecum* extract.

**TABLE 1:** Quantitative phytochemical analysis of aqueous extracts of studied plants.

Phytoconstituents (mg/g)	*Gymnema sylvestre*	*Trigonella foenum‐graecum*
Phenolics	2.5 ± 0.35	4.04 ± 0.20
Flavonoids	9.60 ± 0.30	14.30 ± 0.45
Alkaloids	12.20 ± 0.70	17.70 ± .20
Tannins	14.30 ± 0.50	0.65 ± 0.10
Saponins	25.60 ± 0.40	24.25 ± 0.30

### 3.2 Hypoglycemic effect of *G. sylvestre*, *T. foenum‐graecum*, and metformin

**FIGURE 1:**
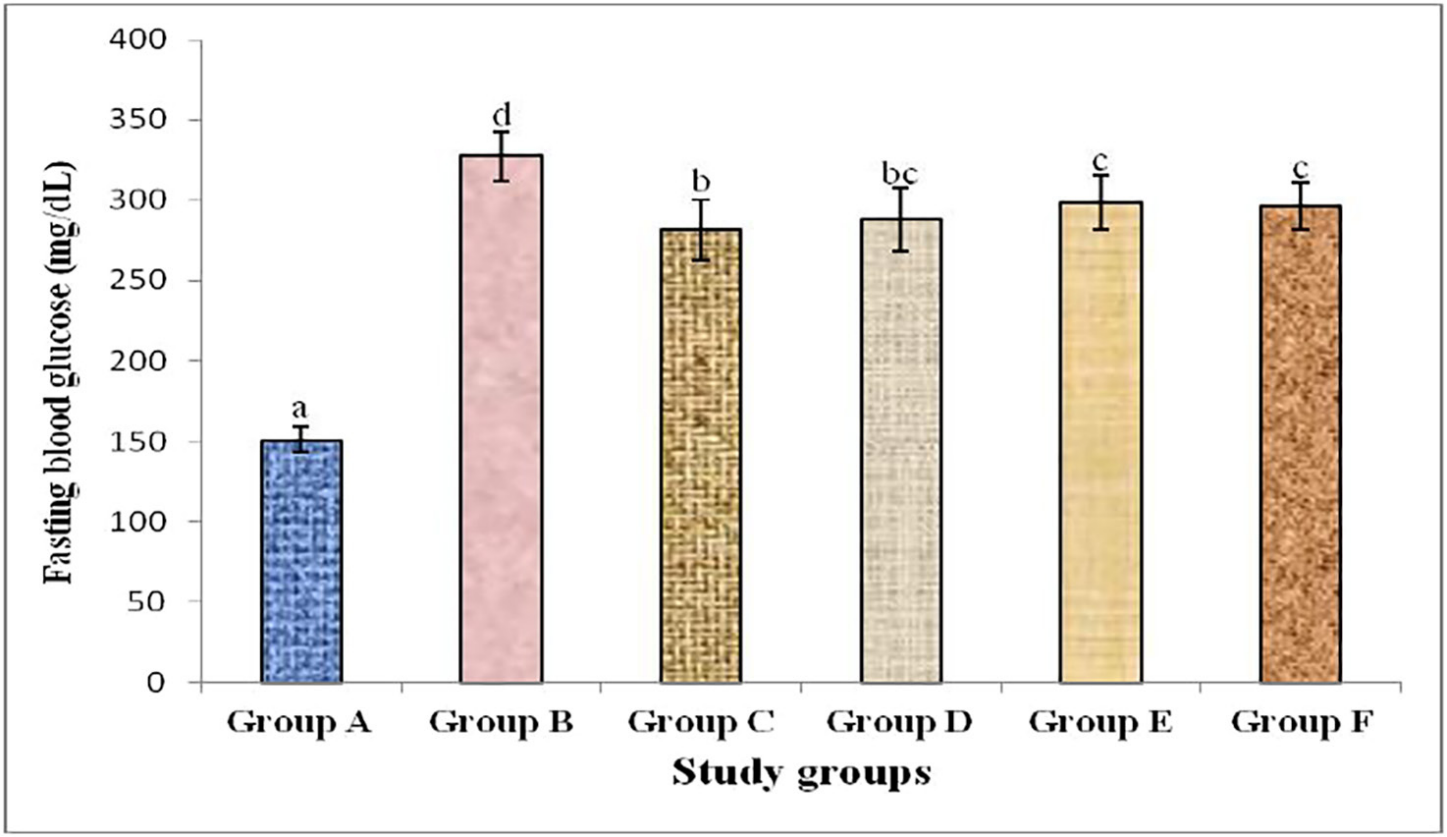
Fasting plasma glucose levels in different study group rabbits showing the effects of plants extract and metformin treatment in alloxan‐induced diabetic rabbits. Different alphabets above different bars indicate significant group mean differences at *p* ≤ .05.

The hypoglycemic effect of *G. sylvestre*, *T. foenum‐graecum*, their mixture and metformin in alloxan‐induced diabetic rabbits is shown in Figure 1. A significant (*p* < .05) decrease in fasting blood glucose level was found in the plants extract‐treated and metformin‐treated rabbit groups as compared to diabetic control group. No significant (*p* > .05) difference was observed between the diabetic‐induced rabbit groups treated with plants extracts and metformin.

### 3.3 Effects on serum insulin level

**FIGURE 2:**
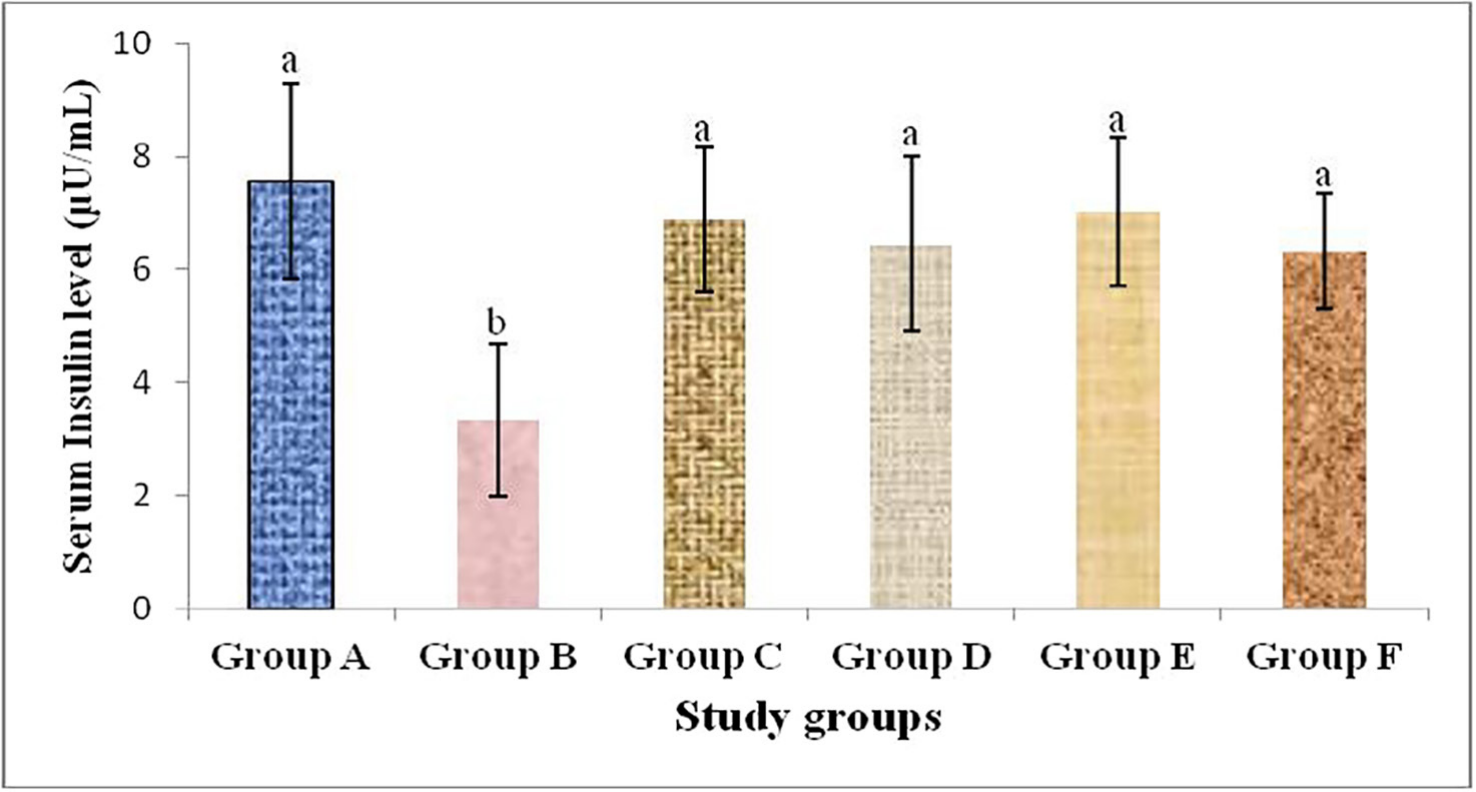
Serum insulin levels in different study group rabbits showing the effects of plants extract and metformin treatment in alloxan‐induced diabetic rabbits. Different alphabets above different bars indicate significant group mean differences at *p* < .05.

Serum insulin level was decreased in alloxan‐induced diabetic rabbits. The current study result showed that serum insulin level was significantly (*p* < .05) increased in alloxan‐induced diabetic rabbits treated with *G. sylvestre*, *T. foenum‐graecum*, their mixture and metformin as compared to diabetic control rabbits. The effect of plants extract to increase serum insulin level in diabetic groups was comparable to metformin‐treated diabetic group rabbits (Figure 2).

### 3.4 Effect on body weight

**FIGURE 3:**
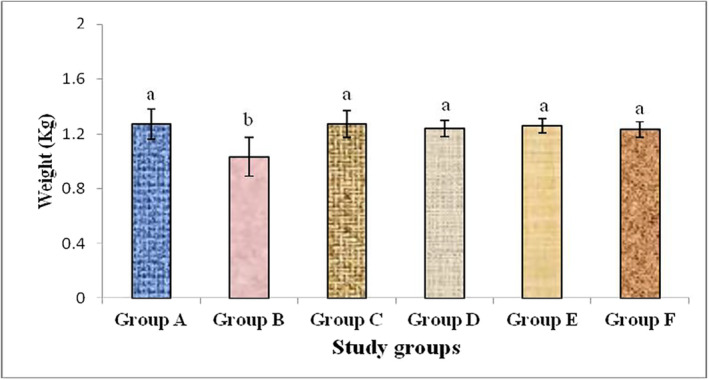
Variation in body weight among different study groups in alloxan‐induced diabetic rabbits treated with studied plants extract and metformin. Different alphabets above different bars indicate significant group mean differences at *p* < .05.

Results of our present study showed that there is marked reduction in the body weight of alloxan‐induced diabetic rabbits. This reduction in the body weight may be attributed to the depletion of insulin, provoking to changes in the metabolism of protein and carbohydrates, and the loss of adipose tissues as a result of diabetic induction by alloxan. Plants extract, their mixture and metformin treatment improved the body weight of all the treatment group rabbits compared to the diabetic control group rabbits. There was significant (*p* < .05) decrease in body weight of diabetic control group rabbits as compared to plants extract and metformin treatment groups. While the difference between metformin and plants extract treated groups was non‐significant (*p* > .05) as shown in Figure 3.

### 3.5 Effect on renal function and PCV

The aqueous extracts of *G. sylvestre*, *T. foenum‐graecum* and their mixture resulted in a significant decrease (*p* < .05) in blood urea level of the treatment group rabbits as compared to diabetic control group rabbits. Metformin also resulted a significant decrease in blood urea level in metformin treated group rabbits as compared to the diabetic control group rabbits. However, there was no significant difference (*p* > .05) between plants extract and metformin treated group rabbits. The aqueous extract of both the plants also decreased the serum creatinine level significantly (*p* < .05) as compared to diabetic control rabbits. Serum creatinine level of extract treated groups was found decreased than metformin treated group. Results of the effect of studied plants extract and metformin treatment in diabetic rabbits are given in Table 2. Alloxan treatment resulted in a significant decrease in PCV. *T. foenum‐graecum* and metformin treatment significantly (*p* < .05) increased the PCV toward normal when compared to diabetic control group rabbits. *G. sylvestre* did not cause a significant effect on PCV compared to diabetic control group rabbits (Table 2).

**TABLE 2:** Effect of aqueous extracts of studied plants and metformin on alloxan‐induced diabetic rabbits.

Parameters/study groups	Group A	Group B	Group C	Group D	Group E	Group F
Urea (mg/dL)	18 ± 4.74^a^	124.2 ± 8.07^d^	43.8 ± 10.18^b^	61 ± 7.81^c^	64 ± 8.87^c^	63.80 ± 8.61^c^
Creatinine (mg/dL)	0.97 ± 0.18^a^	1.95 ± 0.29^d^	1.76 ± 0.28^bc^	1.46 ± 0.61^ab^	1.18 ± 0.27^a^	1.23 ± 0.31^a^
Packed cell volume (%)	38.20 ± 5.11^b^	26.2 ± 2.38^a^	34.60 ± 2.07^b^	29.20 ± 3.03^a^	34.80 ± 3.96^b^	34.2 ± 4.65^b^
Alanine aminotransferase (U/L)	29.2 ± 5.36^a^	96.4 ± 7.16^e^	92.2 ± 4.43^de^	75.60 ± 9.71^b^	85.2 ± 8.34^cd^	77 ± 5.14^bc^
Aspartate aminotransferase (U/L)	62.6 ± 15.53^a^	127.4 ± 10.31^e^	90 ± 19.68^cd^	78 ± 10.36^bc^	78 ± 8.51^bc^	73 ± 7.7bc
Alkaline phosphatase (U/L)	95.8 ± 32.8a	149 ± 9.44c	126.4 ± 10.59b	129.6 ± 9.34^b^	131.4 ± 4.08^b^	130.8 ± 8.16^b^

*Note*: Values are mean ± SD. Different superscript alphabets in rows indicate significant group mean differences among study groups at *p* ≤ .05.

### 3.6 Effect on liver enzymes

The results of the effects of aqueous extracts of plants and their mixture on liver enzymes are given in Table 2. Plants extract and metformin significantly (*p* < .05) decreased the level of ALP toward normal in treatment group rabbits as compared to diabetic control group animals, while ALP level was found increased significantly (*p* < .05) in alloxan‐induced diabetic control animals when compared to normal control group animals. Plants extract treatment significantly reduced the levels of serum ALT and AST in treatment group rabbits as compared to diabetic control group animals, while metformin also causes a mildly significant reduction in serum AST but no effect on serum ALT.

## 4 DISCUSSION

The trend of drug development from the medicinal plants has increased in the recent past and scientists are keenly interested in finding the remedies for challenging maladies from herbs and natural sources. According to Word health organization, 80% of the population in the developing countries relies upon the medicinal plants for their basic health needs against various ailments and 60% of the drugs are derived from medicinal plants (Kauser et al., 2018). Diabetes mellitus is a major problem in the world without any successful treatment. Plants are rich source of bioactive compounds and are being used as alternative medicines because of their potential to treat several diseases (Beressa et al., 2021; Chikowe et al., 2020; Chikowe et al., 2021; Mtewa et al., 2021) including diabetes (Bint Mustafa et al., 2016). There are many synthetic hypoglycemic agents available with several limitations and side effects. To overcome these limitations, exploring the non‐toxic and low‐cost herbal medicines is the need of the hour. Plants such as *T. foenum‐graecum* and *G. sylvestre*, have long history of use as traditional medicine against diabetes are being explored scientifically to develop new anti‐diabetic drugs (Al‐Khateeb et al., 2012; Maurya et al., 2021). Several studies have reported the anti‐diabetic potential of phytomolecules, such as phenolic compounds, flavonoids, saponins, alkaloids, polysaccharides, amino acids, proteins, and coumarins from *T. foenum‐graecum* (Laila et al., 2023). Published studies also reported the anti‐diabetic potential of *G. sylvestre* as the administration of this plant in diabetic‐induced animals resulted in the reduction of blood glucose level, cholesterol, triglycerides, and proteins while increase in HDL‐cholesterol level and insulin (Ahmed et al., 2017; Kumar et al., 2017). Therefore, in this study, the effect of *T. foenum‐graecum*, *G. sylvestre* and their combination along with metformin was studied in alloxan‐induced diabetic rabbits. To the best of our knowledge, this is the first study to investigate and compare the anti‐diabetic potential of both the studied plants extract, their combination and metformin.

Alloxan monohydrate is toxic to insulin producing cells of the pancreas. It causes necrosis of the beta cells which are insulin producing cells through the production of reactive oxygen species (Longkumer et al., 2021). These reactive oxygen species undergo different changes by the Fenton reaction which ultimately results in damage to beta cells of the pancreas and lead to diabetes in experimentally induced animals (Mtewa, 2018; Mtewa et al., 2017 Riaz et al., 2016, 2017; Sathya et al., 2008).

The results of our present study about the lowering effect of fasting blood glucose in *G. sylvestre* treated group rabbits are in agreement with the finding of published study carried out on alloxan‐induced diabetic rats investigating the hypoglycemic effects of *G. sylvestre* (Pothuraju et al., 2014). *T. foenum‐graecum* treatment also resulted in a significant (*p* < .05) decrease in fasting blood glucose level in diabetic rabbits found in our present study is in accordance with the findings of a published study which showed that *T. foenum‐graecum* has significant hypoglycemic effect in diabetic rabbits with mild hypoglycemic effect in non‐diabetic group rabbits (Abdelatif et al., 2012). Whereas, in contrast to this study, ethanolic extract of *T. foenum‐graecum* did not show hypoglycemic effects in either diabetic or non‐diabetic rabbits (Sathya et al., 2008). Serum insulin level was decreased in alloxan‐induced diabetic rabbits. The current study results showed that *G. sylvestre*, *T. foenum‐graecum* and their mixture significantly (*p* < .05) increased the serum insulin level in alloxan‐induced diabetic rabbits as compared to diabetic control group rabbits. Our study results were similar to the findings of Zareen et al. (2008), who reported that medicinal plants increased the serum insulin level in diabetic animals. This effect of plants extract to increase the serum insulin level in diabetic‐induced group rabbits was comparable to the metformin treated group rabbits.

In this study, *G. sylvestre* administration was found to maintain the body weight of rabbits when compared to the body weight of diabetic control group rabbits. Similar studies carried out on rats showed that aqueous extract of *G. sylvestre* resulted in decreased body weight of rats while methanolic extract resulted in increased body weight of rats (Pothuraju et al., 2014). Another study carried out on obese people showed that *G. sylvestre* extract treatment for 8 weeks resulted in a significant decrease in body weight (Preuss et al., 2004). Our study results are also consistent with the findings of another study carried out on alloxan‐induced diabetic rats (Kumar et al., 2012). *T. foenum‐graecum* significantly improved the body weight due to the anti‐oxidant property of this plant which increases the body weight and protects the functional organs of the body (Elghazaly et al., 2019; Fatima et al., 2022).

Alloxan monohydrate results in complications of kidneys indicated by increased level of serum urea and creatinine. *G. sylvestre* treatment in the present investigation resulted in a significant (*p* < .05) decrease in urea and creatinine level in treatment group rabbits as compared to diabetic control group rabbits. The results of our study are in accordance with the findings of previous study carried out in alloxan‐induced diabetic rats (Aslam et al., 2023). Findings of some other studies carried out on streptozotocin‐induced diabetic rats are also in harmony with our results that *G. sylvestre* reduces nephropathy (Kishore & Singh, 2017; Tchouanka et al., 2022). *T. foenum‐graecum* also reduced the level of urea and creatinine compared to positive control group. This is also in agreement with the findings of a published study that reported a reduced serum urea and creatinine levels in *T. foenum‐graecum* extract treated rabbits (Shamim et al., 2016).

Alloxan monohydrate resulted in a significant decrease in PCV in our study. *G. sylvestre* treatment did not show any change in PCV and the findings are in line with an earlier study performed with various doses of *G. sylvestre* which did not change the PCV (Raji et al., 2021). *T. foenum‐graecum* treatment caused a significant (*p* < .05) increase in PCV compared to diabetic control group rabbits. The results of another study showed that *T. foenum‐graecum* fed to goats as 3% of dry matter intake resulted in a significant increase in PCV (Mir et al., 2013).

There are several studies indicating the increased level of hepatic enzymes in diabetic patients. This is observed due to alteration in metabolic processes involving liver enzymes. Level of ALT is increased due to deficiency of insulin and increased activity of amino acids (Sarfraz et al., 2017). *G. sylvestre*, *T. foenum‐graecum* and metformin treatment resulted in a significant decrease in ALP level compared to diabetic control group rabbits. The results of the present study are consistent with the previous study in which *G. sylvestre* returned the level of liver enzymes to normal level in rats (Aly‐Aldin, 2022). Similarly, *T. foenum‐graecum* also resulted in a significant decrease in ALP level compared to diabetic control group animals. Another study have shown that *T. foenum‐graecum* has protective effects against toxicological compounds (Abdel‐Daim et al., 2014). In the present study, *G. sylvestre* extract also caused a significant (*p* < .05) decrease in ALT and AST level compared to diabetic control group and these results comply with the previous study carried on *G. sylvestre* in streptozotocin‐induced diabetic rats normalizing the liver enzymes (El Shafey et al., 2013). *T. foenum‐graecum* also resulted in significant decrease in ALT and AST and these results are in accordance with the previous studies of *T. foenum‐graecum* conducted on biochemical parameters and oxidative stress in rats (Abdel‐Daim et al., 2014; Fatima et al., 2022).

## 5 CONCLUSIONS

It is concluded that methanolic extract of *G. sylvestre* and *T. foenum‐graecum* strongly possessed the anti‐diabetic property. The results of the effects of studied plants are comparable with the anti‐diabetic effect of standard drug metformin. The studied plants extract treatment maintained the body weight, decrease the urea and creatinine level, and increases the activity of liver enzymes in diabetic rabbits. It could be well anticipated that both of these plants have a protectant effect against the kidney and liver damage in diabetics. Further studies are needed to trace out and exploit its active ingredients in anti‐diabetic studies.

## AUTHOR CONTRIBUTIONS

**Muhammad Kashif:** Conceptualization (lead); funding acquisition (lead); investigation (equal); methodology (equal); project administration (equal); supervision (lead); writing – review and editing (equal). **Amar Nasir:** Data curation (equal); formal analysis (equal); investigation (equal); methodology (equal); writing – original draft (equal). **Gulzaman:** Data curation (equal); formal analysis (equal); methodology (equal); visualization (equal); writing – original draft (equal). **Muhammad Kamran Rafique:** Conceptualization (supporting); formal analysis (supporting); methodology (equal); resources (equal); visualization (equal); writing – review and editing (supporting). **Mazhar Abbas:** Conceptualization (supporting); resources (equal); visualization (equal); writing – original draft (equal); writing – review and editing (equal). **Aziz ur Rehman:** Formal analysis (supporting); resources (equal); visualization (supporting); writing – original draft (supporting). **Muhammad Riaz:** Formal analysis (equal); methodology (equal); visualization (equal); writing – original draft (equal); writing – review and editing (lead). **Ghulam Rasool:** Formal analysis (supporting); investigation (supporting); visualization (equal); writing – review and editing (equal). **Andrew G. Mtewa:** Formal analysis (equal); software (equal); visualization (equal); writing – review and editing (equal).

## FUNDING INFORMATION

The funding was obtained under the project (Ref No. 20‐14678/NRPU/cfR&D/HEC/2021) awarded by the Higher Education Commission, Islamabad, Pakistan through National Research Program for Universities.

## CONFLICT OF INTEREST STATEMENT

The authors declare no conflict of interest.

## ETHICS STATEMENT

The study plan was approved by the ethical committee of the University of Veterinary and Animal Sciences, Lahore, Pakistan.

## Data Availability

All data has been provided, however, any other clarifications will be provided upon request.
